# Design and characterization of electroactive gelatin methacrylate hydrogel incorporated with gold nanoparticles empowered with parahydroxybenzaldehyde and curcumin for advanced tissue engineering applications

**DOI:** 10.1007/s10856-024-06808-9

**Published:** 2024-07-29

**Authors:** Zahra Barabadi, Asrin Bahmani, Marzieh Jalalimonfared, Milad Ashrafizadeh, Morteza Rashtbar, Esmaeel Sharifi, Haili Tian

**Affiliations:** 1 Research Center for Molecular Medicine, Hamadan University of Medical Sciences, Hamadan, Iran; 2 Department of Tissue Engineering and Biomaterials, School of Advanced Medical Sciences and Technologies, Hamadan University of Medical Sciences, Hamadan, Iran; 3 Department of Tissue Engineering and Applied Cell Sciences, School of Advanced Technologies in Medicine, Tehran University of Medical Sciences, Tehran, Iran; 4 Department of General Surgery, Institute of Precision Diagnosis and Treatment of Digestive System Tumors, Carson International Cancer Center, Shenzhen University General Hospital, Shenzhen University, Shenzhen, Guangdong 518055 China; 5 International Association for Diagnosis and Treatment of Cancer, Shenzhen, Guangdong 518055 China; 6 Shanghai Institute of Cardiovascular Diseases, Zhongshan Hospital, Fudan University, Shanghai, 200032 China; 7 Department of Radiation Oncology, Shandong Cancer Hospital and Institute, Shandong First Medical University and Shandong Academy of Medical Sciences, Jinan, Shandong 250000 China; 8 Tabriz University of Medical Sciences, Tabriz, Iran; 9 Cancer Research Center, Hamadan University of Medical Sciences, Hamadan, Iran; 10 School of Exercise and Health, Shanghai University of Sport, Shanghai, 200438 China

## Abstract

Electroconductive polymers are the materials of interest for the fabrication of electro-conductive tissues. Metal ions through the redox systems offer polymers with electrical conductivity. In this study, we processed a gelatin methacrylate (GelMA) network with gold nanoparticles (GNPs) through a redox system with parahydroxybenzaldehyde (PHB) or curcumin to enhance its electrical conductivity. Induction of the redox system with both PHB and curcumin into the GelMA, introduced some new functional groups into the polymeric network, as it has been confirmed by H-NMR and FTIR. These new bonds resulted in higher electro-conductivity when GNPs were added to the polymer. Higher electroactivity was achieved by PHB compared to the curcumin-induced redox system, and the addition of GNPs without redox system induction showed the lowest electroactivity. MTT was used to evaluate the biocompatibility of the resultant polymers, and the PHB-treated hydrogels showed higher proliferative effects on the cells. The findings of this study suggest that the introduction of a redox system by PHB in the GelMA network along with GNPs can contribute to the electrochemical properties of the material. This electroactivity can be advantageous for tissue engineering of electro-conductive tissues like cardiac and nervous tissues.

**Graphical Abstract:**

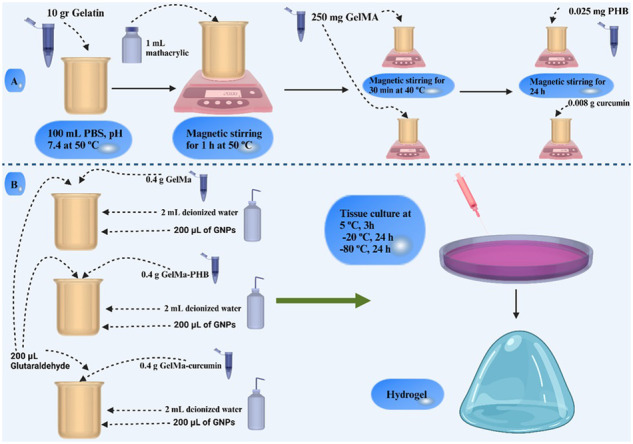

## Introduction

Polymers have greatly contributed to the synthesis of scaffolds for tissue engineering applications [1]. The versatility of polymers allows the utilization of a variety of processing techniques including microfluidics [2], 3D-printing [3, 4], electro-spinning [5], casting [6], and extrusion [7]. Modification of chemical and physical properties of polymers results in different behavior for various applications. For example, conductive polymers have potential applications as bioconductors [8], biosensors [9], nerve and muscle tissue engineering [10], neural conduits [11], and electrically stimulated drug delivery systems [12]. Many conductive polymers, such as polyaniline [13], polythiophene [14], polypyrrole [15], and carbon nanotubes [15], have been used to develop nanofibrous scaffolds.

Polymers are versatile materials with many applications, but their inherent insulating nature limits their use in electroactive tissue engineering [16]. Conductive polymers are suitable materials for regenerating tissues with a natural electrical charge such as cardiac muscle and nervous tissue. Several strategies have been applied to improve the electrical conductivity of polymers. A common method is to add conductive materials to the polymer matrix. These additives can include metals, carbon-based materials like graphene or carbon nanotubes. Modifying polymers with metal nanoparticles is one way to make them conductive [17]. The addition of metal nanoparticles to polymers has expanded the range of characteristics and applications of polymers [18]. Silver and gold nanoparticles (GNPs) are among the most frequently utilized metal nanoparticles for this purpose. In particular, GNP doping in polymers enhances cell-matrix interactions, and leads to an increase in the electrical conductivity of the polymer network [19]. Other characteristics of GNPs that make them attractive for electroactive tissue applications are their antibacterial properties and enhancing the mechanical properties of polymers [20].

Another strategy to improve the conductivity of polymers is to modify the structure of polymer. Redox system is a method that can be used to induce electrical conductivity through the transfer of electrons between two substances. Some polymers possess conjugated structures that allow for the delocalization of electrons, resulting in their electrical conductivity. By doping the polymers with redox-active species, such as dopants or electrolytes, their conductivity can be enhanced, leading to electro activation [19]. The redox-active species in the polymer matrix can undergo reversible redox reactions, meaning they can accept or donate electrons depending on the electrochemical conditions. This redox cycling allows charge transport within the polymer, facilitating the flow of electric current and generating electrochemical reactions. Redox reaction between metal nanoparticles and different moieties of polymeric networks is another method for the induction of electrical conductivity in these materials [21].

Modified polymers such as gelatin methacrylol (GelMA), provide cells with a biological environment favorable for adhesion, growth, and proliferation [22]. GelMA with characteristics such as biocompatibility, biodegradability, and cost-effectiveness has been extensively studied for 3D cell culture applications [23–25]. Similar to gelatin, GelMA has the ability to support cellular activities and the presence of free NH_2_ groups helps to tailor the physical and chemical properties of this polymer according to the needs of different cells and tissues [26].

According to many studies carried out in the field of polymers, the role of functional groups in determining the behavior of polymers is vital. For example, the presence of double bonds, benzene, and heteroatoms can facilitate electron flow through redox reactions in conductive polymers. The synthesis of polymers with a non-toxic molecular structure and antioxidant properties provides the basis for the development of biocompatible polymers [27].

Parahydroxybenzaldehyde (PHB), a compound with redox properties, can serve as a mediator in redox reactions [28]. By incorporating PHB into a polymer matrix, a system can be created in which the polymer undergoes reversible redox reactions. One of the main advantages of using PHB in polymer electrochemistry is its ability to act as both an electron donor and acceptor. This property helps regulate the conductivity and electrochemical properties of the polymer. Through the redox reactions facilitated by PHB, the polymer can switch between different oxidation states, resulting in changes in its charge transport properties [29]. Electrochemical processing of polymers via a redox system using PHB could be a promising avenue to develop functional materials with tunable electrical and electrochemical properties [30, 31]. To create a redox system and optimize the electroactivity of the PHB in the polymer network, specific methods and techniques can be used. These may include the appropriate selection and concentration of PHB, as well as the incorporation of other components or additives to improve the redox properties and overall performance of the material.

Curcumin, a natural compound found in turmeric, has attracted significant attention in recent years due to its potential applications in a variety of fields. Curcumin, with its antioxidant and redox properties can act as a reducing agent, facilitating electron transfer processes and promoting the formation of conductive pathways within the polymer network [32]. One of the main benefits of using curcumin in electrochemical process is its natural origin, making it environmentally friendly compared to synthetic additives. Additionally, curcumin is widely available, cost-effective, and has low toxicity, making it a desirable choice for various applications [33].

**Fig. 1:**
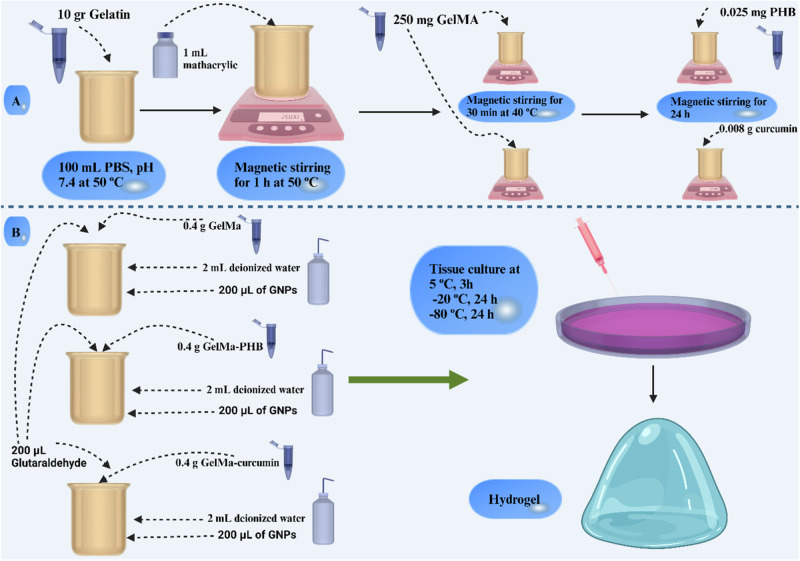
**A** First step is the synthesis of GelMA. 10 gr gelatin was mixed in 100 mL PBS and then, 1 mL methacrylic was added during magnetic stirring for 1 h. Then, two following steps were performed to synthesis GelMA-PHB and GelMA-curcumin; **B** The second step is the synthesis of hydrogel performed by adding GelMA, GelMA-PHB, and GelMA-curcumin separately with deionized water and GNPs. Finally, they underwent tissue culture and the hydrogel was constructed

In this study, new redox-conducting polymers based on GelMA, plus GNPs and PHB or curcumin were synthesized. The conductive properties of GelMA were expected to be improved through redox reaction between the functional groups of the modified matrix and by the addition of GNPs. Figure 1 schematically demonstrates the synthesis of hydrogels.

## Material and methods

### Chemistry

All chemicals and solvents were purchased from Merck and Sigma-Aldrich companies. IR spectra of products were recorded on KBr disks by the Alpha-BRUKER IR apparatus. The ^1^H-NMR spectra were recorded by a Bruker (500 MHz, D_2_O) spectrometer.

### General procedure for the synthesis of GelMA

**Scheme 1:**
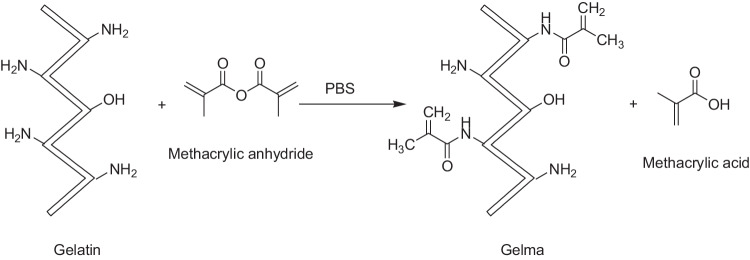
Synthesis of GelMA

GelMA synthesis was performed according to Van Den Bulcke et al. procedure [34]. 10 g of gelatin (type A) was dissolved in 100 mL of phosphate buffer saline (PBS, pH 7.4) at 50 °C. When the reaction mixture became clear, 1 mL of methacrylic anhydride was added to the reaction mixture under magnetic stirrer conditions. The reaction continued for 1 h at 50 °C. In order to remove byproducts and impurities, the reaction mixture was poured into a dialysis bag and dialyzed for 4 days at room temperature. After, the reaction products were frozen and freeze-dried during 3 days. The synthetic route for the GelMA is depicted in Scheme 1.

### General procedure for the synthesis of GelMA-PHB

**Scheme 2:**
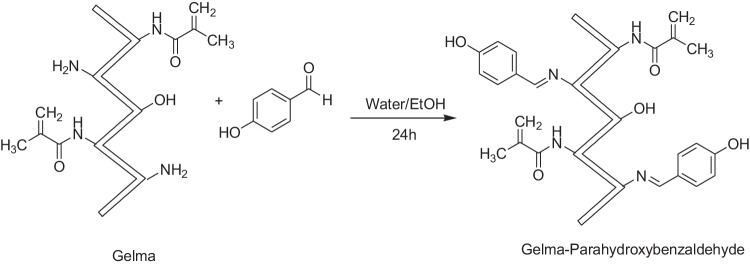
Synthesis of GelMA-PHB

In a 50 mL round-bottom flask, GelMA (250 mg) and 5 mL deionized water were stirred at 40 °C for 30 min under N_2_ atmosphere. Then, PHB (0.025 g) was dissolved in 3 mL ethanol and were added dropwise to reaction mixture. The reaction was heated at 40 °C for overnight under continuous stirring. After 24 h, to purify the product, the reaction mixture was dialyzed in 0.5 L ethanol by dialysis tubing with cutoff 2000 Da for 24 h. Ethanol was removed to some extent under reduced pressure. The crude polymer was dissolved in water and lyophilized to dryness and stored in a desiccator at 4 °C. NMR and FT-IR spectroscopy techniques were used to confirm the structure of GelMA-PHB. The synthetic route for GelMA-PHB is depicted in Scheme 2.

### General procedure for the synthesis of GelMA-curcumin

**Scheme 3:**
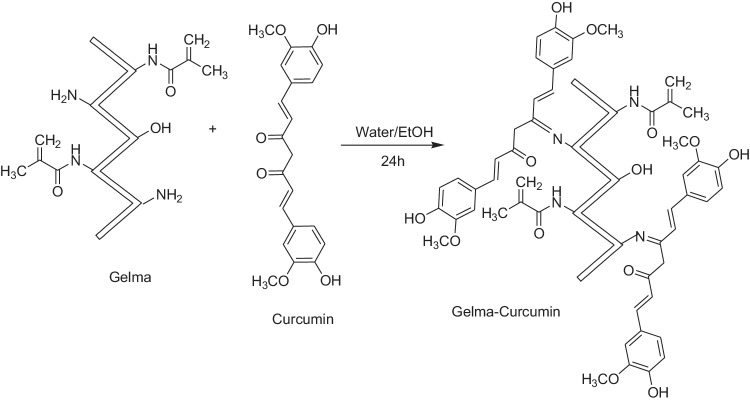
Synthesis of GelMA-curcumin

The synthesis steps are the same as for GelMA-PHB, except for 0.008 g of curcumin that was used instead of PHB. The synthetic route for GelMA-curcumin is depicted in Scheme 3.

### Hydrogel preparation

0.4 g of GelMA, GelMA-PHB, and GelMA-curcumin were dissolved separately in 2 ml of deionized water (20% (w/v)). 200 µL of GNPs were added to each solution in order to achieve GelMA-GNP, GelMA-PHB-GNP and GelMA-curcumin-GNP, respectively. In the next step, 20 µL of glutaraldehyde (1% (w/v)) was used to crosslink the polymers. Gel-freeze casting method was used to form cross-linked polymers [17]. The samples were poured separately into tissue culture plate using a syringe. The samples were kept at 5 °C for 3 h, then at −20 °C for 24 h and finally at −80 °C for 24 h. Ethanol was used to remove the excess amount of glutaraldehyde and the samples were washed four times with deionized water. Samples were lyophilized and stored in freezer for further experiments.

### Assessment of conductivity

GelMA, GelMA-GNP, GelMA-PHB-GNP and GelMA-curcumin-GNP hydrogels were swollen in PBS buffer (pH 7.4). The solutions were poured onto a Petri dish for the hydrogel casting at the room temperature. Two probes made contact with the surface of each film sample. The probes were connected to the apparatus (Keithley, Model 6517A) for a constant voltage of 12 V. The applied voltage and the resultant current were used to determine the electrical conductivity of the polymer. Pure PBS solution was tested as control.

### In-vitro cytocompatibility assessment

In order to evaluate the biocompatibility of the synthesized polymers, the hydrogels were casted in 96 well plates and following stepwise freezing in −20 for 4 h and −80 for 16 h, freeze drying for 24 h was performed in order to have porous structures. MTT assay was performed on human adipose mesenchymal stem cells. Briefly, 3000 cells on their third passage were seeded on the UV-sterilized hydrogels. 72 h following cell culture in DMEM with 10% FBS and 1% PENSTREP, and incubation in 37 °C, 10 μl of MTT was added to each well. After 3.5 h, the supernatant was discarded and 100 μl DMSO was added to solubilize the formazan crystals. 5 min incubation in DMSO, the supernatant was transferred to another 96 well plate and the absorbance was measured at 570 nm with Microplate reader. Un-modified GelMA was considered as control.

## Results and discussion

### Chemistry

GelMA, GelMA-PHB, and GelMA-curcumin were synthesized and identified by the ^1^H NMR and IR spectrum. The chemical shifts of ^1^H NMR spectrum of gelatin, GelMA, GelMA-PHB, and GelMA-curcumin in 4 ranges of 9–10, 6–9, 3–6, and 0–3 ppm are presented in Table 1. Indicator peaks in each category are shown in red, which will be discussed below.

**Table 1:** The chemical shifts of ^1^H NMR spectrum of gelatin, GelMA, GelMA-PHB, and GelMA-curcumin

δ (ppm)	Gelatin	GelMA	GelMA-PHB	GelMA-curcumin
9–10	–	–	9.64	–
6–9	7.15	6.86	6.93	7.14
	7.22	7.03	6.96	7.90
	8.34	7.21	7.03	8.28
			7.20	
			7.77	
			7.80	
			7.95	
			8.31	
			8.32	
3–6	3.09	3.11	3.11	3.07
	3.23	3.24	3.66	3.51
	3.52	3.52	3.76	3.61
	3.82	3.80	3.85	3.72
	3.84	3.91	3.91	3.81
	4.04	3.93	4.24	3.86
	4.23	4.25	4.67	4.19
	4.51	5.32		4.28
		5.56		
0–3	0.75	0.82	0.82	0.09
	0.81	1.11	1.11	0.78
	1.10	1.29	1.29	1.07
	1.24	1.31	1.56	1.09
	1.28	1.57	1.71	1.13
	1.31	1.80	1.81	1.24
	1.55	1.92	1.92	1.52
	1.91	1.95	2.26	1.76
	2.17	2.25	2.62	1.85
	2.59	2.72		1.92
	2.88			2.07
				2.20
				2.57

**Fig. 2:**
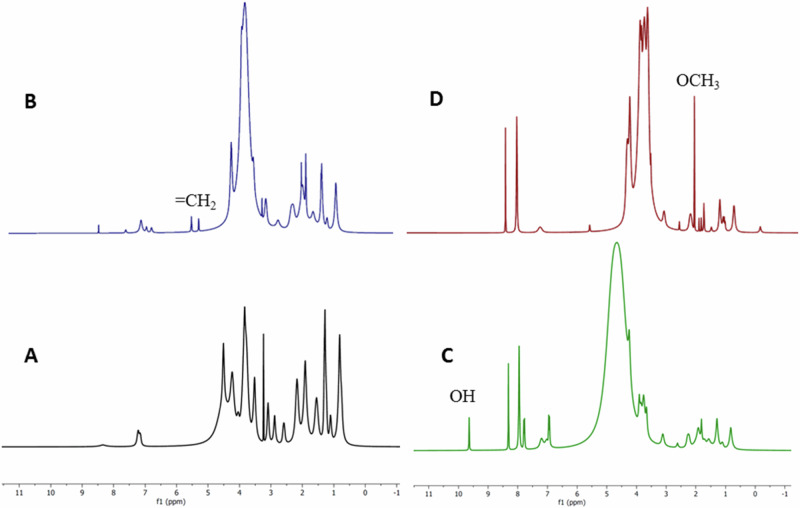
1H NMR spectrum of (**A**) gelatin, (**B**) GelMA, (**C**) GelMA-PHB, and (**D**) GelMA-curcumin

Figure 2 shows ^1^H NMR spectrum of gelatin, GelMA, GelMA-PHB, and GelMA-curcumin. It can be observed that the signals at δ = 5.32 and 5.56 ppm are attributed to double bound protons. These signals are not observed in the ^1^H NMR spectrum of gelatin, so it is a sufficient reason for the synthesis of GelMA (Fig. 2A, B). The ^1^H NMR spectrum of GelMA-PHB is in the range of δ = 0.8–9.64 ppm (Fig. 2C). It is crystal clear that the signal at δ = 9.64 ppm is attributed to hydroxyl proton. Peaks in the range δ = 7.77–8.32 ppm are related to PHB aromatic protons. The peaks of the double bond protons are not visible in the range of δ = 5–6 ppm due to the broadening of the peaks. The ^1^H NMR spectrum of GelMA-curcumin is in the range of δ = 0.7–8.28 ppm (Fig. 2D). The presence of signals δ = 7.9 and 8.28 ppm can be attributed to the aromatic protons. Interestingly, the signal corresponding to the protons of the methoxy group at δ = 2.07 ppm is quite clear and sharp. The peaks of the hydroxyl group and the protons of the double bonds are hidden due to the broad peaks of the GelMA.

### IR spectroscopy

**Fig. 3:**
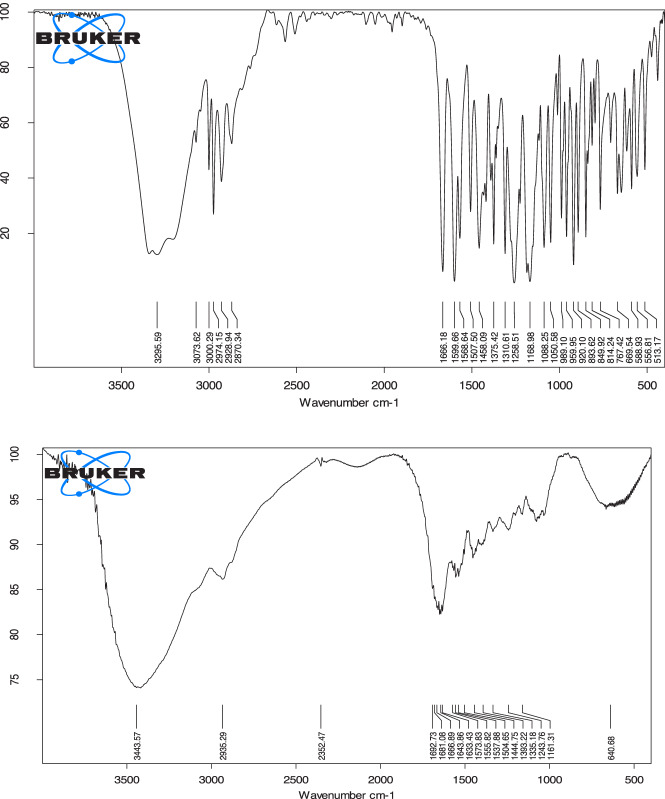
Representative IR spectrum of Top) GelMA and Down) Gelatin

**Fig. 4:**
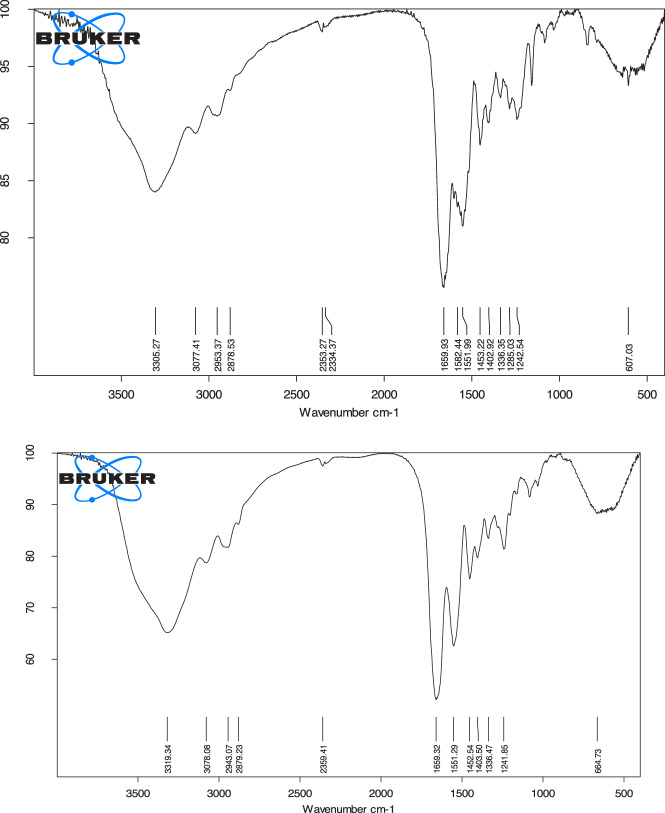
Representative IR spectrum of Top) GelMA-PHB and Down) GelMA-curcumin

According to the molecular structure of gelatin, GelMA, GelMA-PHB and GelMA-curcumin, functional groups OH and NH_2_ are common to them, and their distinguishing feature in the IR spectrum is the presence of carbonyl, C-H aromatic and C = N groups. According to Fig. 3 (Top), of GelMA carbonyl groups, absorption band having shifted to 1666 cm^−1^, gelatin carbonyl groups appear at 1692 cm^−1^. The shift of stretching vibration of carbonyl groups from 1692 to 1666 cm^−1^ indicates the presence of conjugated carbonyl amide groups with a double bond. All in all, carbonyl amide groups are present in GelMA-PHB and GelMA-curcumin, the shift of the peak is observed very partial (Fig. 4). The broadening of the carbonyl peaks of GelMA-PHB and GelMA-curcumin is very urgent, which indicates the existence of imine groups (C=N) and conjugated ketone group with a double bond. The presence of peaks in the region of 3000–3170 cm^−1^ (C-H stretching vibration) indicates the addition of aromatic rings to polymers modified with PHB and curcumin (Fig. 4).

### Hydrogel preparation

**Fig. 5:**
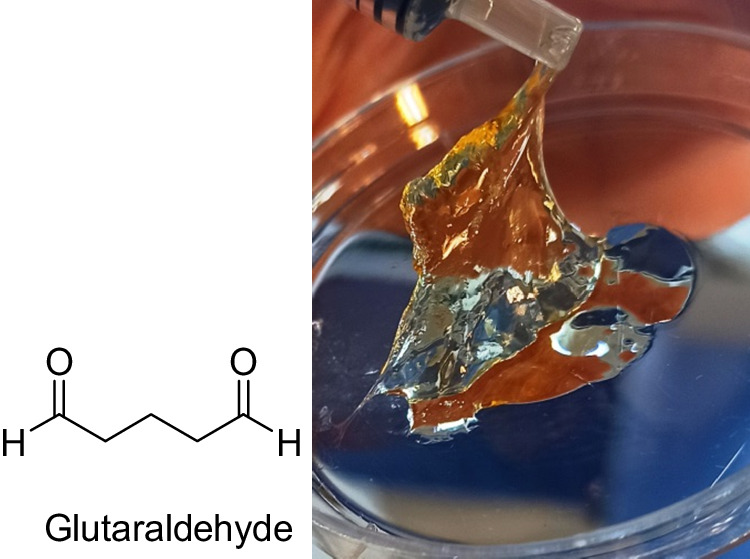
Shows the gel and cross-linked form of GelMA-PHB-GNP

Considering the structure of GelMA-PHB and GelMA-curcumin and the presence of aromatic rings in the branches, linkers with a suitable length should be used. Due to the structure of glutaraldehyde (Fig. 5), it was considered a suitable linker for this category of polymers.

### Conductivity

The data of the conductivity experiment are presented in Table 2. The highest conductivity is attributed to GelMA-PHB-GNP and the lowest to GelMA. The higher conductivity of GelMA-GNP compared to GelMA is related to the carbonyl groups and double bonds that can participate in electron flow with GNPs. The presence of benzene in GelMA-PHB-GNP and their electronic connection with GNPs has led to maximum conductivity in this study. The increase in the number of benzene, double bonds, and carbonyl groups in GelMA-curcumin-GNP has caused disorder in the electron flow network; as a result, the conductivity has decreased compared to GelMA-PHB-GNP.

**Table 2:** Electro-conductivity of swollen hydrogels

Sample	Conductivity (s/cm)
GelMA	0.118
GelMA-GNPs	0.127
GelMA-PHB-GNPs	0.217
GelMA-Curcumin-GNPs	0.168

### Biocompatibility

**Fig. 6:**
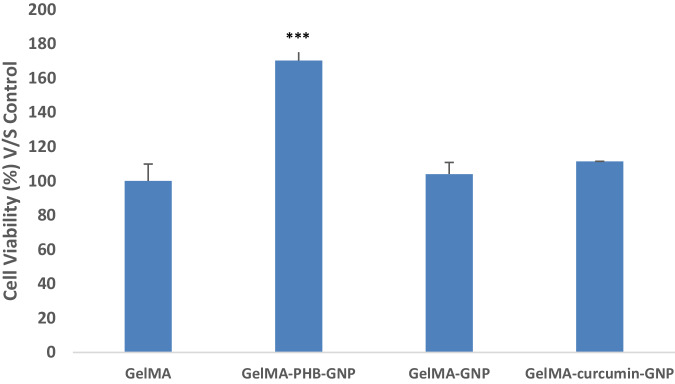
Cell viability of hMSCs on GelMA, electroconductive GelMA with PBH and gold nanoparticles (GelMA-PBH-GNP), electroconductive GelMA with gold nanoparticles (GelMA-GNP), and electroconductive GelMA with Curcumin and gold nanoparticles (GelMA-curcumin-GNP). Higher cell viability and proliferation was achieved with GelMA-PHB-GNP

Interestingly, GelMA modification in all cases resulted in higher rate of biocompatibility (Fig. 6). Electroconductivity through PHB treatment significantly increased the rate of cell viability over all non-treated and treated GelMA. Although addition of GNPs to GelMA, alone or following curcumin treatment increased the rate of cell viability, but it was not statistically significant in compared to GelMA alone.

## Discussion

Tissue engineering of electrically active tissues is an exciting field that combines principles of biology, engineering, and materials science to create functional tissues capable of electrical activity. This research area is very promising for applications in conductive tissue engineering [35]. The goal of tissue engineering is to develop functional replacements for damaged or diseased tissues. In the case of conductive tissues, such as cardiac or nervous tissue, an important goal is to reproduce their unique electrical properties [36]. One approach in tissue engineering of electro-conductive tissues involves the use of conductive biomaterials. These materials, such as conductive polymers, can provide a scaffold for cell attachment and growth while also facilitating electrical communication between cells. By incorporating these materials into three-dimensional scaffolds, it is possible to create an environment that supports the development of functional electroactive tissues [37]. In 2021, Zhu et al. designed and synthesized an injectable conductive hydrogel based on GelMA/oxidized dextran/reductive graphene oxide matrix as cell carriers of umbilical cord mesenchymal stem cells (UCMSCs) for myocardial infarction (MI) repair. It was shown that the designed conductive hydrogel has a high potential for repairing cardiac tissue [38].

The design and synthesis of redox-active species that are compatible with the polymer matrix and exhibit desirable electrochemical properties can be complex. The selection of appropriate dopants or electrolytes requires careful consideration of their redox potential, solubility, and compatibility with polymers [39].

In this study, we showed that the modification of GelMA using both PHB and curcumin led to the introduction of new carbonyl amide and benzene groups into the GelMA network as it has been shown in the H NMR spectrums and confirmed by the FTIR experiment. It indicates the successful incorporation of the redox system into the polymer network. Functionally, it is supported by the data from electroactivity experiment, where we found that modification of GelMA using both PHB and curcumin led to higher conductivity. Modification of GelMA with PHB along with GNPs doping showed the highest conductivity rate and GNPs doping into unmodified GelMA showed the lowest conductivity after unmodified GelMA. This implies that although the addition of GNPs can increase the electroactivity, electrical activation using a redox system amplifies this effect; and in this respect, PHB is more effective than curcumin. The use of PHB and GNPs in polymer electro activation opens up possibilities for multifunctional materials. In a study by ref. [40], PHB was used as a precursor for synthesizing phenolic polymers with tunable electrical conductivity properties through the formation of redox-active polymer composites. Through PHB polycondensation with formaldehyde, the obtained polymer exhibited a low but non-negligible electrical conductivity, in the range of 0.09–2.97 μS/cm. It was supposed that the conductivity arised from the polarization of the carbonyl group in the polymer backbone, which facilitates charge separation and transport along the conjugated system [40].

In another study by Zhang, it has been shown that the incorporation of gold nanoparticles significantly enhanced the electrical conductivity of polymers like PEDOT:PSS. This enhancement was attributed to effective electron transfer facilitated by the porous polymer network, as well as synergistic interactions at the metal-polymer and metal-metal interfaces [41].

It is worth mentioning that in other studies, curcumin has been used to improve the properties of hydrogels. In ref. [42] used methacryloyl hydrogels modified with curcumin to reduce the reactive oxygen species-induced adipose-derived stem cell apoptosis and improve wound healing. The results of their study showed that the antioxidant properties of hydrogels modified with curcumin improved and the final hydrogels can be effective as biological materials in healing diabetic wounds. The interesting thing about their study is that their goal was not to covalently bind curcumin to the methacryloyl polymer and it was limited to the loading and absorption of curcumin in the hydrogels [42].

Another study that used curcumin as an enhancer of nanoparticle properties was conducted by ref. [43] They developed a novel anti-inflammatory composite hydrogel scaffold with curcumin encapsulated in solid lipid nanoparticles and mixed it with gelatin methacrylate (GelMA) hydrogel to treat intervertebral disc degenerative disease (IDD). The results of their study showed that the restoration of Collagen type II was done by hydrogels modified with curcumin, in turn resulting the restoration of intervertebral discs. In their study, covalent bonding between curcumin and polymer was not considered [43].

Combining electro-activity with other desirable properties, such as biocompatibility, could lead to the development of advanced materials for tissue engineering applications. Induction of electroactivity using three different methods in this study was shown to result in increased polymer biocompatibility, although the highest cell proliferation rates was achieved for the cells on PHB modified GNPs doped GelMA.

The results of this study showed that both PHB and curcumin can introduce functional groups or redox-active molecules that can undergo reversible oxidation and reduction reactions into the GelMA polymer network, leading to electroactivity. Additionally, doping Gelma with GNPs and forming composites can further enhance their electrical conductivity. This electroactivity could be advantageous for a variety of applications, such as tissue engineering of electro-conductive tissues like cardiac and nervous tissue.

The molecular structure, especially the presence of conjugated systems and functional groups with capacity of redox reactions can affect the polymer conductivity. In our experiment, we embedded the GNPs into GelMA hydrogels with the introduction of PHB and curcumin as redox agents to promote the electroactivity of the materials. The lowest conductivity was related to GelMA lacking any modification (0.118 s/cm). The poor coducitivity results from the HOMO of GelMA that is positioned around the gelatin backbone with restricted conjugation to reduce electron delocalization. The localization of LUMO is also similar that can mediate the insulting nature of GelMA. The addition of GNPs improved the conductivity of GelMA (0.127 s/cm) because of the presence of metal nanostructures can provide more conductive pathways. The HOMO and LUMO in the GNP-embedded GelMA, there is a light shift towards the lower energy, highlighting the slight improvement in the delocalization of electron and conductivity. GelMA-PHB-GNP hydrogel has shown the highest conductivity (0.217 s/cm) that partially is related to the function of PHB in the addition of redox-active functional groups in improving the eletron delocalization. The HOMO level showed high delocalization in this composite, while LUMO demonstrated lower level, highlighting the favorable electron acceptance and increase in conductivity. Similar to us, Ramalakshmi and colleagues demonstrated that same improvements through the application of redox-active polymers [28]. The GelMA-curcumin-GNP hydrogel demonstrated a moderate conductivity (0.168 s/cm) that appears higher than GelMA-GNP, but lower than GelMA-PHB-GNP. In spite of the function of curcumin in the addition of some redox-active sites, its capacity is lower than PHB. In this regard, HOMO showed partial delocalization over curcumin moieties, but LUMO was decreased not that significant compared to PHB-modified hydrogel. Our results are also in line with the results of ref. [40] about the polymer conductivity affected by the conjugated systems [40].

## Conclusion

The induction of redox systems in polymers for electro activation refers to the process of introducing and controlling redox reactions within polymers to enable electrochemical activation. This can be achieved by incorporating redox-active molecules or functional groups into the polymer matrix, which can undergo reversible oxidation and reduction reactions. The present study showed that the redox system introduced by PHB is more effective than curcumin in inducing electroactivity in GelMA and the addition of GNPs further enhances this property. The redox system involving PHB and GNPs can provide a platform for the electrochemical process of polymers. PHB can undergo reversible oxidation and reduction reactions, and GNPs, in turn act as an electron mediator for these redox reactions, facilitating the electron transfer process between PHB and the polymer matrix. This promotes effective redox processes, leading to improved electrochemical performance of the polymer. The electrochemical processing of Gelma through the redox system offers several advantages. First, it allows tuning the electrical properties of the Gelma by controlling the PHB and GNPs concentrations. Additionally, the combination of PHB and GNPs can enhance the stability and durability of the electroactive Gelma. This system also supports cell survival and proliferation for tissue engineering applications. However, challenges still exist in this field. Optimization of the redox system, including the selection of appropriate concentrations and ratios of PHB and GNPs, requires further research. Additionally, the long-term stability and compatibility of these materials with different polymer matrices need to be thoroughly investigated.

### GelMA-PHB

1H NMR (301 MHz, Deuterium Oxide) δ 9.64 (s, 2H), 8.31 (d, J = 2.0 Hz, 3H), 7.95 (s, 2H), 7.78 (d, J = 8.1 Hz, 2H), 7.41–6.83 (m, 7H), 4.67 (s, 25H), 4.24 (s, 4H), 3.88 (d, J = 17.2 Hz, 9H), 3.76 (s, 5H), 3.11 (s, 5H), 2.62 (s, 3H), 2.26 (s, 3H), 1.92 (s, 1H), 1.29 (s, 1H), 0.82 (s, 1H).

### GelMA-curcumin

1H NMR (301 MHz, Deuterium Oxide) δ 8.28 (s, 1H), 7.90 (s, 2H), 7.14 (s, 3H), 5.51 (s, 1H), 4.28 (s, 4H), 4.19 (s, 4H), 3.84 (d, J = 15.8 Hz, 10H), 3.72 (s, 6H), 3.61 (s, 6H), 3.07 (s, 1H), 2.20 (s, 1H), 2.07 (s, 1H), 1.92 (s, 1H), 1.85 (s, 1H), 1.76 (s, 1H), 1.52 (s, 1H), 1.24 (s, 1H), 1.17–1.04 (m, 2H), 0.78 (s, 1H).

### GelMA

1H NMR (301 MHz, Deuterium Oxide) δ 7.36–6.77 (m, 7H), 5.58 (d, J = 14.1 Hz, 2H), 5.32 (s, 2H), 4.25 (s, 5H), 3.94–3.88 (m, 7H), 3.80 (s, 7H), 3.18 (d, J = 37.7 Hz, 4H), 2.66 (d, J = 30.6 Hz, 3H), 2.25 (s, 1H), 1.92 (s, 2H), 1.57 (s, 1H), 1.30 (d, J = 7.5 Hz, 2H), 0.82 (s, 2H).

### Gelatin

1H NMR (301 MHz, Deuterium Oxide) δ 7.22 (s, 1H), 4.54–4.48 (m, 3H), 4.23 (s, 2H), 4.04 (s, 1H), 3.84–3.79 (m, 3H), 3.52 (s, 2H), 3.23 (s, 1H), 3.09 (s, 1H), 2.88 (s, 1H), 2.59 (s, 1H), 2.17 (s, 2H), 1.91 (s, 3H), 1.55 (s, 2H), 1.29 (d, J = 7.3 Hz, 5H), 1.10 (s, 1H), 0.81 (s, 3H), 0.75 (s, 2H).
